# Regulation of aquaporin-2 using traditional Chinese medicine in water balance disorders: a literature review

**DOI:** 10.3389/fcvm.2025.1506190

**Published:** 2025-09-23

**Authors:** Yifan Chang, Lu Liu, Xiaodong Xu, Shiqiang Zhang

**Affiliations:** ^1^ The Graduate School, Heilongjiang University of Chinese Medicine, Harbin, China; ^2^ Hebei Province Key Laboratory of Integrated Traditional and Western Medicine in Neurological Rehabilitation, Hebei Province Cangzhou Hospital of Integrated Traditional Chinese and Western Medicine, Cangzhou, Hebei, China

**Keywords:** Aquaporin-2, traditional Chinese medicine, water balance disorders, cardiovascular diseases, Zhenwu decoction

## Abstract

Aquaporin-2 (AQP2) is a critical protein involved in water metabolism. It is primarily located in the renal collecting duct cells’ apical plasma membranes and intracellular vesicles and regulates the movement of water into and out of cells. It plays a pivotal role in maintaining fluid balance, and its dysregulation is associated with conditions such as hypertension and heart failure, which contribute to cardiovascular disease progression. AQP2 has garnered significant attention as an emerging therapeutic target. Traditional Chinese medicine (TCM) has demonstrated efficacy in treating water balance disorders, although its underlying mechanisms remain elusive. The discovery of AQP2 and its association with water metabolism provides an opportunity for TCM to explore these mechanisms more intuitively. This review integrates TCM formulas, single herbs, and active constituents, linking them to AQP2 regulation in the kidneys, heart, liver, inner ear, and uterus with an emphasis on the AVP–V2R–AQP2 axis, while distinguishing between short- and long-term regulation and highlighting cardiovascular applications. This review synthesizes the current evidence from experimental and limited clinical studies, highlights the regulatory effects of TCM on AQP2 in various organ systems, and identifies key research gaps to guide future translational and clinical investigations.

## 1 Introduction

Aquaporins (AQPs) are a family of membrane proteins that are specifically responsible for facilitating water transport across cellular membranes, with some subtypes permitting the passage of small molecules such as glycerol and urea (1). To date, at least 13 AQPs (AQP0–AQP12) have been identified in mammals. These proteins are distributed across various organs and tissues, significantly enhancing water permeability in the cellular membrane and performing critical physiological functions through their involvement in intracellular and extracellular water balance regulation (2). Among these, aquaporin-2 (AQP2) is predominantly localized in the apical plasma membrane and intracellular vesicles of renal collecting duct cells. AQP2 is uniquely responsive to arginine vasopressin (AVP), facilitating water transport in and out of renal cells, thereby enhancing water reabsorption from urine (3). Due to its critical role in renal water balance regulation, AQP2 dysfunction is closely associated with various kidney pathologies.

Recent studies have revealed that, beyond renal function, AQP2 is also involved in water metabolism disorders related to other organ systems, notably the cardiovascular system, liver, inner ear, and uterus (4). In particular, emerging evidence highlights the distinctive role of AQP2 in cardiovascular diseases through its modulation of fluid balance and blood pressure regulation, underscoring the importance of exploring its potential as a therapeutic target.

Traditional Chinese medicine (TCM) has historically provided comprehensive diagnostic and therapeutic approaches to managing conditions arising from abnormal water metabolism, such as pathological states involving water dampness and phlegm fluid retention (5). Clinical practice demonstrates that TCM interventions frequently yield substantial therapeutic effects when used to treat these disorders. At the molecular level, the function of AQP2 is primarily regulated through the vasopressin type 2 receptor (V2R) signaling pathway. In this classical pathway, AVP binds to V2R located on the basolateral membrane of renal collecting duct principal cells, leading to the activation of adenylate cyclase (AC) via Gs protein signaling (6). This activation increases intracellular cyclic adenosine monophosphate (cAMP) levels, which subsequently activate protein kinase A (PKA). Activated PKA then promotes the phosphorylation and trafficking of AQP2-bearing vesicles to the apical membrane, thereby increasing water reabsorption by enhancing membrane water permeability (6).

At present, TCM research is increasingly focused on this signaling pathway, providing novel insights and therapeutic targets for managing water metabolism disorders. This review aims to systematically summarize the current research regarding TCM interventions that modulate AQP2 expression and activity in diseases related to water metabolism. Moreover, we aim to elucidate the underlying molecular mechanisms, thereby providing robust theoretical support and guidance for future clinical applications of TCM in treating water metabolism disorders.

## 2 Literature search method

Relevant studies were identified through systematic searches of multiple databases, including PubMed, Web of Science, Embase, China National Knowledge Infrastructure (CNKI), and Wanfang. The search terms used were combinations of “Aquaporin-2,” “AQP2,” “Traditional Chinese Medicine,” “TCM,” “water metabolism disorders,” “renal diseases,” “cardiovascular diseases,” “edema,” and the names of specific Chinese medicinal formulas and herbs known to regulate water metabolism. Articles published within the past 20 years were selected based on their relevance to the regulatory effects and mechanisms of TCM on AQP2 expression and activity. In addition, references from key articles were reviewed to ensure comprehensive coverage of the relevant literature.

Most of the included studies comprised *in vivo* animal models (primarily rats and mice) and explored the regulatory effects of TCM formulas or compounds on AQP2 and related aquaporins in pathological conditions such as heart failure, renal dysfunction, and edema.

## 3 Regulatory effects on kidney function and mechanisms

### 3.1 Zhenwu decoction

AQP2 is the most critical aquaporin in renal tubular epithelial cells, which play a crucial role in renal tubule concentration and reabsorption. In renal diseases, edema and oliguria are typical manifestations of fluid metabolism disorders according to TCM theory, and TCM treatment has the benefits of few side effects and remarkable curative effects (7). Zhenwu decoction, a classical formula for treating edema in TCM, has good clinical efficacy (8). Animal experiments have proved that Zhenwu decoction has an effect on the renal function of rats with renal failure and alleviates their water metabolism disorders by raising serum AVP levels, downregulating AQP2 protein expression in the renal tissues of spontaneously hypertensive rats (SHR), and lowering the level of AQP2 in urine (9). Scholars suggested that Zhenwu decoction may operate similar to AVP, which activates the “AVP-V2R-AQP2” pathway, and proposed that a drug-containing serum of Zhenwu decoction may increase the expression of V2R or prolong the time that V2R stays on the surface of the basement membrane, continuously binding to receptors and ultimately increasing the presentation of AQP2, thereby regulating the metabolism of water by improving the water permeability (10, 11).

As the main component of Zhenwu decoction, *Poria cocos* (Fuling) has been found harbor total triterpene, water-soluble polysaccharides, and water-insoluble polysaccharides, which increased the expression of AC and PKA, and decreased the expressions of antidiuretic hormone (ADH), V2R, AQP1, and AQP2 in edema in lower energizer model rats with renal deficiencies in recent studies (12, 13). It is speculated that the increase in serum ADH content initiates V2R and inhibits the action of AC. Thus, the intracellular concentrations of cAMP and PKA decrease, leading to an increase in AQP2, which improves renal water reabsorption (14). This conclusion supported the previous speculation that the chief components of *Poria cocos* resemble aldosterone (ALD) receptor antagonists.

### 3.2 Shenqi Wan

Shenqi Wan also contains *Poria cocos*, which can rectify water metabolism disorders in renal deficiency. In consideration of the “multi-component, multi-target” action characteristic of compound formulae in TCM, scholars utilized the relevant inhibitors and agonists to intervene in each link of the “V2R-cAMP-AQP2” pathway and confirmed that Shenqi Wan may have an effect similar to AVP, which activates the AVP-V2R-AQP2 pathway through V2R (15). In addition, PKA is also one of the significant targets of Shenqi Wan, adjusting AQP2 expression. Compared with Zhenwu decoction, Shenqi Wan has more complex components and combinations. It has been previously demonstrated that *Rhizoma alismatis* (Zexie), another fundamental component of Shenqi Wan, has the effect of inhibiting the expression of AQP2 in the renal medulla of normal rats (16).

### 3.3 Zhuling decoction

Zhuling decoction, which also contains *Poria cocos* and *Rhizoma alismati*, was found to downregulate AQP2 mRNA and protein expression, increase urine volume in rats with adriamycin nephropathy (AN), and improve water and sodium metabolism in nephrotic syndrome (17). Ergosterol, the index component of *Polyporus umbellatus* (Zhuling), has also been found to have a critical diuretic effect and markedly downregulate the mRNA and protein expressions of AQP1, AQP2, and AQP4 (18, 19).

### 3.4 Er Shen Wan

Er Shen Wan has been found to diminish polyuria symptoms through hormone regulation (20). Moreover, Er Shen Wan has been found to improve renal water absorption and increase AQP2 and arginine vasopressin receptor 2 (AVPR2) expression levels (21). However, these studies were limited by experimental conditions and time; thus, the effects of Er Shen Wan on AVPR2 and AQP2 protein expression should be further confirmed, and more detailed analyses of the regulatory mechanism are needed. The composition of the TCM compound is complicated, so it is essential to conduct an in-depth study on its composition and the principles of its combination and compatibility in order to clarify the internal mechanism and material basis of its therapeutic effect.

## 4 Application of single Chinese herbs in water metabolism disorders

Studies have demonstrated that a single Chinese herb can also be effective in treating water metabolism-related diseases. Urine production is regulated by renal autoregulation and humoral regulation, and urine volume chiefly depends on the amount of water reabsorbed by distal convoluted tubules and the renal collecting duct. This is affected by changes in the aquaporin expression in this segment. The humoral factors involved in the regulation of urine production are manifold, among which ADH, ALD, and atrial natriuretic peptide (ANP) are the most significant.

### 4.1 *Phytolacca acinosa* (Shanglu)

A study discovered that *Phytolacca acinosa* Roxb (Shanglu), when processed with vinegar, can significantly decrease the AQP2 expression in rat kidneys, act as a vasopressin-sensitive water channel, significantly decrease serum ADH and ALD, and increase the content of ANP (22). There is speculation that the regulating effect may be associated with further changes in the regulation of water channel protein expression levels by kidney hormones.

### 4.2 Lagopsis supina

*Lagopsis supina* and its four soluble parts were found to have potential diuretic activity in salt-loaded rats and significantly inhibited renin angiotensin aldosterone system (RAAS) activity, including lowering serum angiotensin II (Ang II), ADH, and ALD levels and increasing serum ANP levels (23). In particular, two components of *Lagopsis supina*, stachysoside A and acteoside, have practical diuretic effects through the inhibition of AQPs and RAAS and upregulation of atriopeptin expression in salt-loaded rats, which strongly supports the potential use of *Lagopsis supina* as a new diuretic (24). Previous studies have revealed that Ang II may play a meaningful role during AQP2 transport to chief cells of the medullary collecting duct (25). Further quantitative research needs to be explicit about whether AQP-2 is directly related to RAAS in regulating urine volume.

### 4.3 Quercetin

In chronic renal failure (CRF), TCM can also play an adjunctive role by regulating AQP2. Researchers have shown that quercetin, the main component of Shengqing Jiangzhuo capsule, may regulate AQP1 and AQP2 in the kidneys of rats with chronic renal failure, thereby moderating water retention and toxin accumulation to relieve CRF (26).

### 4.4 *Ephedra* (Mahuang)

*Ephedra* (Mahuang), a water-draining and swelling-dispersing medicinal herb, has a therapeutic effect in renal edema. Experts found that a water extract of *Ephedra* and its alkaloid components can inhibit the expression of AQP1 and AQP2 in rat kidneys. They speculate that *Ephedra* and its alkaloid fractions may lower the expression of AQP1 and AQP2 to achieve the effect of inducing diuresis to alleviate edema (27). In further research, a water extract of *Ephedra* and its alkaloid components increased the urine volume of rats and reduced the plasma ADH level and the expression of AQP2 protein in the kidneys. This research concluded that its diuretic effect may be related to the level of ADH. However, a different result was observed in another experiment, as a water extract of *Ephedra* and its alkaloid components had no effect on AQP1 and AQP2 in the kidneys of a rat model with a water retention pattern in the upper energizer (28). This contradiction with previous research results may be attributed to the differences in the models, as they focused on the kidneys that belonged to the lower energizer and the upper energizer, respectively.

### 4.5 *Epimedium* (Yinyanghuo)

In addition to renal AQP2, *Ephedra* and *Epimedium* (Yinyanghuo) can suppress the expression of AQP3 in the lungs for the treatment of edema, while a high dose of *Epimedium* alone can significantly reduce the levels of AQP2 in the kidneys (29, 30), regulating the secretion of relevant body fluids from various organs of rats under water load to induce diuresis. This conclusion is consistent with the theory that *Ephedra* enters the lung meridian and *Epimedium* enters the kidney meridian, which may explain the insignificant effect of *Ephedra* on renal AQP2 in some specific disease models. This series of studies indicated that the regulatory effect of traditional Chinese medicine on AQP2 may be related to medicinal meridian tropism and pattern identification, including visceral pattern identification, triple energizer pattern identification, and six-meridian pattern identification in TCM, which may guide studies on Chinese medicinal targets in the future.

## 5 Regulatory effects of traditional Chinese medicine on water metabolism in various organ systems

### 5.1 Regulating effects on hypertension

Water and sodium metabolism disorders in the kidneys are the leading mechanisms of salt-sensitive hypertension. Wulingsan has a great effect when used to treat hypertension resulting from water metabolic disorders in the kidneys. Researchers have shown that Wulingsan can significantly relieve water reabsorption by decreasing the expression of AQP2 protein and AVP-V2R in kidney tissue from rats with hypertension, preliminarily illustrating the hypotensive mechanism of Wulingsan on a molecular level (31). Containing *Polyporus umbellatus*, *Poria cocos*, and *Rhizoma alismatis*, Wulingsan has a similar regulation mechanism to Zhuling decoction, proving the reliability of the study (32). Scholars have demonstrated that acupuncture with Zusanli (ST36) and Quchi (LI11), combined with acupoint application on Pishu (BL20), regulates the abnormal expression of AQP1 and AQP2 in the kidneys, thus correcting the water metabolism disorder to decrease blood pressure and control the occurrence and progression of hypertension (33). Studies on AQP1 and AQP2 will provide new ideas for elucidating the pathogenesis of salt-sensitive hypertension. Acupuncture combined with acupoint application can modulate the expression of AQP1 and AQP2, which may be one of the mechanisms by which acupuncture lowers blood pressure, with great significance for the prevention and treatment of hypertension.

### 5.2 Regulating effects in chronic heart failure

Vasopressin is considered the main mechanism of water retention in chronic heart failure (CHF) (34). During heart failure, the sensitivity of the atrial stretch receptors decreases, giving rise to a weakened inhibition effect on AVP, which increases the concentration of AVP in circulating blood. AQP2 is then transferred from the cytoplasm to the membrane through the AVP-V2R-AQP2 pathway, resulting in water retention in the body by increasing water reabsorption (35). The water retention resulting from the abnormal increase in AQP2 is a fundamental cause of the poor prognosis of CHF. Therefore, AQP2 has attracted attention as an emerging target in research into water metabolism imbalance and has become an entry point for the treatment of cardiac edema (Table 1).

**Table 1:** Regulation of aquaporin expression by traditional Chinese medicine in cardiovascular diseases.

Type	Distribution	Function	Associated diseases	Traditional medicine	Aquaporin affected	Associated cardiovascular effects	Reference
AQP1	Found in the kidneys, eyes, brain, lungs, etc.	Facilitates water transport across cell membranes, especially in renal tubules	Hypertension, kidney diseases, CHD, eye disorders	Yiqi Jianpi formula	AQP1↓	Downregulates NF-κB, IL-6, and TNF-α and AQP1 expression in myocardial tissue, reducing inflammatory response and edema, and thereby improving coronary heart disease symptoms	(36)
				Naoxintong capsules	AQP1↓	Inhibits AQP1 expression, alleviates myocardial edema and cell apoptosis, and exerts cardioprotective effects	(37)
				XinLi formula	AQP1↓	Alleviates myocardial fibrosis by inhibiting AGTR1/NLRP3 signaling, reducing plasma IL-1β, IL-18, IL-6, and TNF-α levels; mitigates myocardial edema by suppressing the interaction between AGTR1 and AQP1	(5)
AQP2	Primarily in renal collecting ducts	Regulates water reabsorption, crucial for maintaining water balance	Kidney diseases, heart failure, hypertension, hyponatremia	Bushen Huoxue recipe	AQP2↓	Decreases serum BNP in CHF rats and downregulates AQP2 in renal tissue, thereby improving cardiac function	(38)
				*Descurainiae sophia*	AQP2↓	Targets the RAAS and AVP systems to reduce AQP2 expression in renal tissue, increases urine output, and thus improves heart failure	(39)
				Wenyang Xiaoying	AQP2↑	Reduces serum BNP levels in a CHF rat model and upregulates renal AQP2	(40)
AQP3	Found in the kidneys, skin, gastrointestinal tract, and lungs	Participates in water reabsorption and glycerol transport	Xerosis, skin hydration, cardiac toxic injury, and kidney diseases	*Ganoderma lucidum*	AQP3↓	Downregulates AQP3, AQP4, and AQP7 levels, improving myocardial cell injury	(41)
AQP4	Found in the brain, spinal cord, and eyes	Regulates cerebrospinal fluid and water balance in the brain	Brain edema, chronic pulmonary heart disease, multiple sclerosis	Jianxin Pinglv pills	AQP4↑	Reduces the incidence and mortality of reperfusion arrhythmias and alleviates ischemic myocardial edema injury	(42)
				Jian Xin soups	AQP4↑	Inhibits the inflammatory response, improving cardiac function and blood oxygenation	(43)
				Tongxinluo capsules	AQP4↓	Inhibits AQP1, AQP4, AQP8, and AQP9 expression, reducing myocardial edema and ameliorating myocardial cell injury	(44)
AQP7	Found in adipose tissue, liver, and kidneys	Primarily responsible for glycerol and water transport	Diabetes, obesity, cardiac toxic injury	Yiqi Wenyang Zhushui soups	AQP7↓	Improves heart failure status, enhances ATP levels in failing hearts, and downregulates AQP1, AQP4, and AQP7	(45)
				Xinshuitong capsule		Suppresses AQP1, AQP4, and AQP7 expression, alleviating myocardial edema and cardiac remodeling, thereby mitigating the cardiotoxicity of antineoplastic agents	(46)
AQP8	Found in the liver, kidneys, and pancreas	Facilitates water and glycerol transport	Liver diseases, myocardial infarction, and pancreatic disorders	Tongxinluo capsule	AQP8↓	Inhibits AQP1, AQP4, AQP8, and AQP9 expression, reducing myocardial edema and alleviating myocardial cell injury	(44)
AQP9	Found in the liver and white blood cells	Regulates water and glycerol transport	Liver diseases, myocardial infarction, and immune system disorders	Tongxinluo capsules	AQP9↓	Inhibits AQP1, AQP4, AQP8, and AQP9 expression, reducing myocardial edema and alleviating myocardial cell injury	(44)

NF-κB, nuclear factor kappa B; IL-6, interleukin 6; TNF-α, tumor necrosis factor alpha; CHF, chronic heart failure; CHD, coronary heart disease.

There are two regulatory mechanisms of AQP2, namely short-term and long-term regulation. According to the differences in their principles, scholars have carried out studies on the effects of drugs on AQP2. It has been reported that the Yiqi Huoxue formula inhibits the increase in AQP2 protein expression induced by long-term continuous elevated AVP levels, in which cAMP/exchange protein is directly activated by the cyclic adenosine monophosphate (Epac)/extracellular signal-regulated protein kinase (ERK) signaling pathway involved in long-term regulation (47). Experiments have shown that the short-term application of water-draining medicinal herbs [*Poria cocos*, plantain seeds (Cheqianzi), and pepperweed seeds (Tinglizi)] decreases AQP2 expression in the urine of rats with heart failure, and the conclusion is consistent with previous studies (48). However, the effect of blood-activating medicinal herbs [*Radix salviae miltiorrhizae* (Danshen), *Carthamus tinctorius* (Honghua), and *Radix paeoniae rubra* (Chishao)], qi-tonifying medicinal herbs [*Radix ginseng rubra* (Renshen) and *Radix astragali* (Huangqi)], and interior-warming medicinal herbs [*Cassia* twig (Guizhi) and *Aconite* (Fuzi)] was not obvious under the same conditions. With increasing administration time, the effects of the latter three sorts of drugs on AQP2 continuously increased, while the impact of water-draining medicinal herbs did not change significantly. *R. salviae miltiorrhizae* is associated with a reduction of AQP2, leading to a redistribution of AQP2 in renal collecting ducts (49). Moreover, the therapeutic effect of *R. astragali* on heart failure was partly related to an improvement in the AVP system and the abnormal expression of AQP2 in rats. The difference in the results may be related to the short-term and long-term regulatory mechanisms of different drugs, which are worthy of further study.

**Figure 1:**
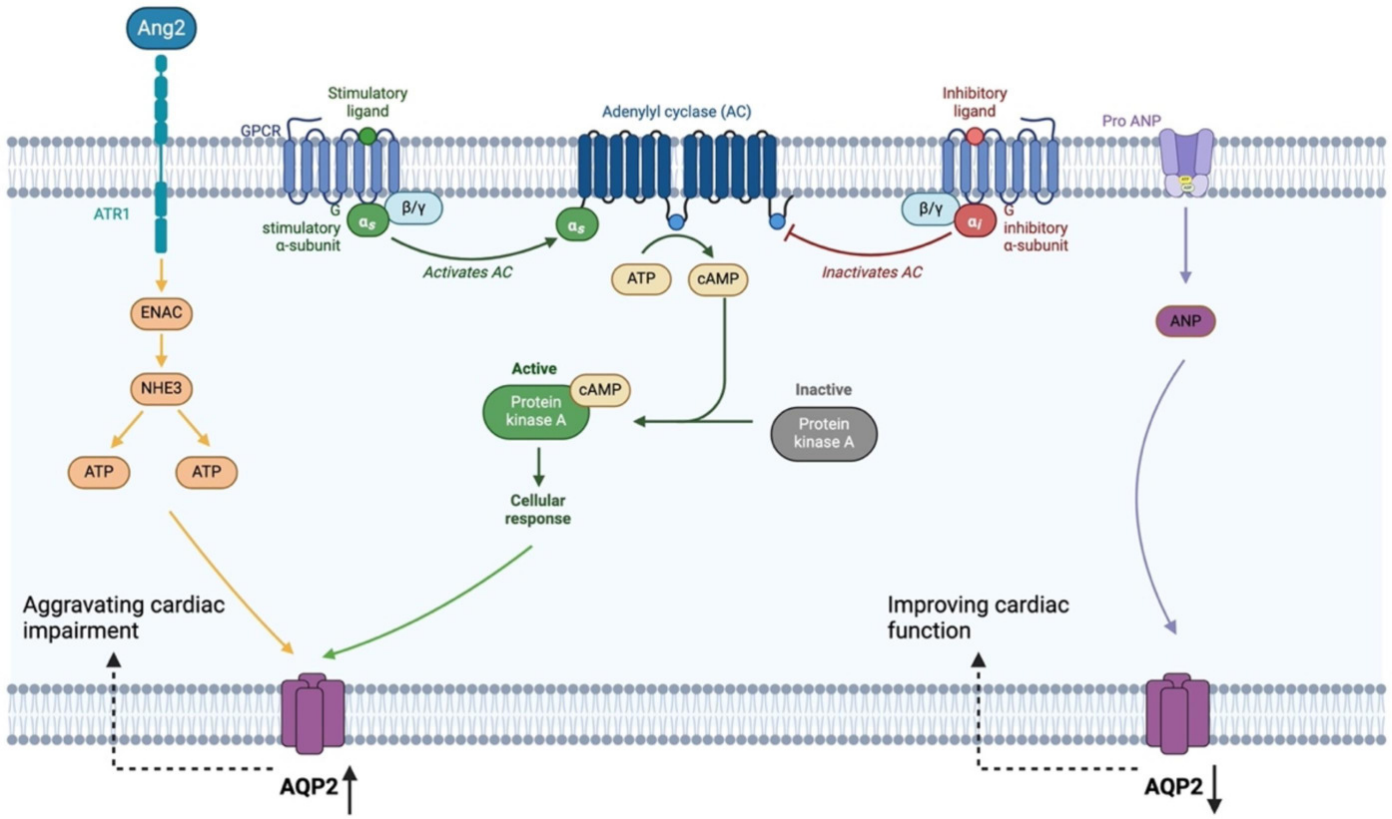
Mechanisms by which traditional Chinese medicine regulates AQP2 in water metabolism disorders. AQP2-mediated regulation of renal water balance is intimately linked to the development and progression of heart failure. Angiotensin II (Ang II) engages AT1 receptors to trigger the ENaC/NHE3 pathway, leading to VP release and increased aldosterone production. These hormonal signals synergistically elevate AQP2 expression in renal collecting duct cells, resulting in fluid retention and hyponatremia that worsen heart failure symptoms. In contrast, the serine protease Corin converts pro-ANP into its active form, ANP, which suppresses AQP2 expression, and ultimately improving the clinical manifestations of heart failure.

In addition, the Bushen Huoxue recipe was found to decrease the level of BNP in serum and AQP2 expression in renal tissue of CHF model rats, and its regulatory effect was similar to captopril tablets (38). Therefore, it can be inferred that the Bushen Huoxue recipe may be used to treat CHF by lessening the expression of AQP2 in the kidneys, but this needs to be confirmed by further experimental data (Figure 1).

### 5.3 Regulating effects on the liver

Ascites is one of the complications of liver cirrhosis and hepatocellular carcinoma. In decompensated cirrhosis, increased portal venous pressure leads to renal insufficiency and water retention, resulting in ascites that seriously affects the patient's quality of life (50). AVP and AQP2 are thought to play an essential role in ascites formation. It has been proven that when the expression of AQP2 and V2R is downregulated, liver ascites in mice with hepatocellular carcinoma is significantly alleviated and ascites symptoms are relieved. Danggui Shaoyao San may decrease the expression of AQP2 to treat ascites by inhibiting the release of AVP and the binding of AVP and V2R (51). Scholars found that the protein expression levels of AQP2 and V2R were considerably increased in a hepatocellular carcinoma rat model with ascites after 12 days of ascites inoculation, while *Euphorbia pekinensis* (Daji) decoction effectively moderated abnormally elevated AQP2 and V2R expression levels (52). The inhibitory effect of *E. pekinensis* was further enhanced when combined with twice the amount of *Glycyrrhiza* (Gancao). Preliminary mechanism exploration verified that *Glycyrrhiza* combined with *E. pekinensis*, when used to treat ascites in hepatocellular carcinoma, may regulate renal AQP2 and V2R (53). Researchers have found that 3-O-(2'E,4'Z-decadienoyl)-20-O-acetylingenol (3-O-EZ), a major diterpenoid of EK, exhibits a specific therapeutic effect on ascites in mice with hepatoma (54). In conclusion, AQP2 inhibition may be a therapeutic strategy for treating ascites in hepatoma.

### 5.4 Regulating effects on the inner ear

In a study on water metabolism-related diseases of the inner ear, all the downstream factors of AVP in the AVP-V2R-AQP2 system that regulate AQP2 expression were found to be present in the inner ear. The AQP2 protein is mainly distributed in the stria vascularis, Corti's organ, and spiral ganglion cells (55). Previous animal experiments showed that the classical Linggui Zhu Gan decoction and Zexie decoction formulae lower the degree of endolymphatic hydrops induced by intraperitoneal injections of desmopressin acetate in guinea pigs (56). One of the mechanisms may be the downregulation of AQP2 expression in the vestibular membrane. Electroacupuncture may modulate the AVP-V2R-AQP2 system and lower the expression of AQP2 in the whole body, which in turn decreases the opening of water channels to weaken endolymphatic hydrops symptoms. However, electroacupuncture at GV20 can reverse the decrease in V2R expression caused by AVP (57). Whether the effect of electroacupuncture on V2R expression is directly caused by electroacupuncture or due to the decrease in plasma AVP still needs to be studied further.

### 5.5 Regulating effects on the uterus

AQP2 expression is periodic as it is upregulated in the endometrial epithelium during the secretory phase and plays a vital role in the periodic changes of the endometrium (58). Shaofu zhuyu decoction was found to decrease the AQP1 and AQP5 mRNA levels and upregulate the AQP2mRNA expression level in the abdominal fluid of rats with endometriosis (EM), thus affecting the disease course (59). In this case, the drug works by restraining the promotion of AQP2 to endometrial debris infiltration, which may be a function of AQP2. AQP2 expression in the vaginal epithelium at different stages of fertility may play a role in regulating the production and quantity of vaginal secretions. Clinical research indicates that the use of TCM treatment after cervical columnar epithelium ectopic physical therapy can alleviate an increase in postoperative vaginal secretions and abnormal water drainage and shorten the vaginal drainage duration (60). Moreover, scholars found that AQP2 expression diminished simultaneously after TCM treatment, indicating that there was an apparent connection between the decreasing expression of AQP2 and the decrease in vagina drainage.

## 6 Impact of traditional medicines on the metabolism of cardiovascular drugs

In addition to modulating AQP2 expression to regulate water metabolism, TCM can also influence the pharmacokinetics and pharmacodynamics of conventional cardiovascular drugs, including diuretics and antihypertensive agents. Several TCM formulations and single herbs possess active compounds that interact with key regulatory pathways, such as the renin-angiotensin-aldosterone system (61) and the sympathetic nervous system, both of which are critical in blood pressure regulation and fluid balance. For instance, herbs such as *Ephedra* and *Glycyrrhiza* have been shown to affect not only water reabsorption mechanisms via AQP modulation but also the activity of cytochrome P450 enzymes involved in drug metabolism (62). Such interactions alter the bioavailability or efficacy of diuretics and hypertensive medications, potentially leading to synergistic effects. When TCMs are used in conjunction with diuretics, by targeting different points (such as AQP2 expression and ion channels/pumps), they can enhance the excretion of water and electrolytes, potentially increasing the diuretic effect and aiding in the alleviation of edema or hypertension caused by sodium and water retention (63–65). However, combined use, if not properly matched in dose or formula, can increase risks such as electrolyte disturbances and dehydration, necessitating close monitoring of patient electrolyte levels. However, some TCMs that have tonic effects or regulate kidney yang may promote water reabsorption under certain stages or specific pathogenic conditions, which could theoretically reduce the efficacy of diuretics if combined inappropriately. Thus, a comprehensive consideration of patient diagnosis and pharmacological properties is required.

Moreover, by modulating neurohormonal pathways, TCM enhances the therapeutic outcomes of conventional therapies or reduces their side effects, offering a complementary approach in managing complex conditions such as heart failure and hypertension (66). Given these possibilities, further research is warranted to systematically explore the molecular mechanisms behind these interactions, which may ultimately facilitate the rational design of integrated treatment regimens that combine TCM with standard cardiovascular drugs for optimized patient care.

## 7 Limitations and future prospects

This study primarily focused on the regulation of AQP2 by traditional Chinese medicine and its application in diseases related to water metabolism. However, there are still limitations that warrant further exploration and improvement. First, most current research on the regulation of AQP2 by TCM primarily involves *in vitro* cellular studies and animal experiments and lacks large-scale, high-quality randomized controlled clinical trial data. Due to differences between *in vivo* environments and actual clinical conditions, the extrapolation and reproducibility of experimental results in clinical settings still require further validation. Second, compound traditional Chinese medicines often contain multiple herbs, characterized by their multicomponent and multitarget synergistic effects. Although existing studies suggest that these effects may be mediated through the AVP-V2R-AQP2 signaling pathway or other AQP subtypes, the specific drug–receptor binding modes, downstream signaling networks, and the mechanisms of interaction between components lack systematic and in-depth elucidation. AQP2's regulation is distinguished into short-term and long-term mechanisms at the molecular level; for example, short-term regulation primarily involves the trafficking and localization of AQP2, whereas long-term regulation is associated with gene transcription and protein synthesis. Current research on the effects of TCM over different durations on AQP2 is insufficient, making it difficult to comprehensively assess the optimal timing for intervention and the sustainability of effects at various stages of disease. Although this article focuses on the pivotal role of AQP2 in water metabolism, other AQPs (such as AQP1, AQP3, and AQP4) also play significant roles in fluid balance across various tissues and organs. Whether interventions with TCM synergistically or adversely affect the expression or function of other AQPs has been minimally studied and warrants further exploration. The application of TCM emphasizes individualized treatment based on differential diagnosis; coupled with the fact that the quality of Chinese medicinal materials, preparation techniques, and methods of administration have not yet been completely standardized, this may lead to variations in clinical efficacy and safety across different studies or regions. Future efforts are required to continually improve the quality control of Chinese medicines and standardize clinical dosing. Although there are some exploratory studies suggesting potential synergistic or antagonistic interactions when TCM is used with diuretics and antihypertensive drugs, the current research is not systematic and lacks rigorously designed pharmacokinetic and pharmacodynamic trials and conclusive clinical evidence.

## 8 Conclusions

As can be seen from the above studies, AQP2 is a critical protein in the regulation of water metabolism and has become a valuable target in the study of TCM treatment of water metabolism imbalance. However, as AQPs are widely distributed in the body, it should be considered whether TCM affects the expression of other aquaporins while regulating AQP2. At the same time, the relationship between the effect of TCM on AQP2 expression and neural and humoral regulation requires further research. Moreover, TCM has fewer side effects and can be used for a long duration. However, due to the lack of detailed research on long-term and short-term regulation of AQP2, the limits of its effect are not clear, and the specific role of TCM still requires further study. In conclusion, an in-depth study on the mechanism of AQP2 and its influence on diseases may provide new ideas for TCM treatment of related diseases (Supplementary Table S1).
